# A Machine Learning Model to Predict Postoperative Speech Recognition Outcomes in Cochlear Implant Recipients: Development, Validation, and Comparison with Expert Clinical Judgment

**DOI:** 10.3390/jcm14113625

**Published:** 2025-05-22

**Authors:** Alexey Demyanchuk, Eugen Kludt, Thomas Lenarz, Andreas Büchner

**Affiliations:** 1 Department of Otorhinolaryngology, Hannover Medical School, Carl-Neuberg-Straße 1, 30625 Hannover, Germany; 2 Hearing4all Cluster of Excellence, Hannover Medical School, 30625 Hannover, Germany

**Keywords:** cochlear implantation, machine learning, predictive modeling, hearing loss, clinical decision-making

## Abstract

**Background/Objectives**: Cochlear implantation (CI) significantly enhances speech perception and quality of life in patients with severe-to-profound sensorineural hearing loss, yet outcomes vary substantially. Accurate preoperative prediction of CI outcomes remains challenging. This study aimed to develop and validate a machine learning model predicting postoperative speech recognition using a large, single-center dataset. Additionally, we compared model performance with expert clinical predictions to evaluate potential clinical utility. **Methods**: We retrospectively analyzed data from 2571 adult patients with postlingual hearing loss who received their cochlear implant between 2000 and 2022 at Hannover Medical School, Germany. A decision tree regression model was trained to predict monosyllabic (MS) word recognition score one to two years post-implantation using preoperative clinical variables (age, duration of deafness, preoperative MS score, pure tone average, onset type, and contralateral implantation status). Model evaluation was performed using a random data split (10%), a chronological future cohort (patients implanted after 2020), and a subset where experienced audiologists predicted outcomes for comparison. **Results**: The model achieved a mean absolute error (MAE) of 17.3% on the random test set and 17.8% on the chronological test set, demonstrating robust predictive performance over time. Compared to expert audiologist predictions, the model showed similar accuracy (MAE: 19.1% for the model vs. 18.9% for experts), suggesting comparable effectiveness. **Conclusions**: Our machine learning model reliably predicts postoperative speech outcomes and matches expert clinical predictions, highlighting its potential for supporting clinical decision-making. Future research should include external validation and prospective trials to further confirm clinical applicability.

## 1. Introduction

Hearing loss is one of the leading global health challenges and a significant cause of disability, particularly among older adults [1]. According to the World Health Organization (WHO), over 1.5 billion people worldwide experience some degree of hearing loss, and this number is projected to increase due to demographic shifts and noise exposure [2]. Untreated hearing loss hinders day-to-day interactions and substantially diminishes quality of life, often leading to social isolation, depression, and cognitive decline in older adults [3].

Cochlear implantation (CI) is well-established as a highly effective treatment method for individuals with severe-to-profound sensorineural hearing loss, offering significant improvements in speech perception, communication abilities, and overall quality of life [4,5]. However, postoperative outcomes are not uniformly beneficial; some recipients derive greater hearing gains than others due to a host of variables such as age at implantation, etiology of hearing loss, cognitive status, and neural survival [6,7]. Consequently, clinicians, researchers, and patients alike have long sought accurate methods for predicting CI outcomes—both to optimize patient selection and to establish realistic expectations in preoperative counseling.

Numerous approaches have emerged to address this need, many of which rely on preoperative patient clinical data (e.g., audiometric thresholds, aided speech recognition scores, demographics) [8,9,10]. Key prognostic factors that have frequently been identified include age at implantation, duration of profound hearing loss, postlingual onset, and duration of hearing aid use. Moreover, newer approaches using machine learning (e.g., random forests, decision trees, support vector machines) have shown promise in uncovering complex interactions among predictive variables [11,12,13]. Such models hold potential to assist clinicians in deciding candidacy for CI and to improve patient counseling by providing individualized outcome estimates. One multi-center effort by Shafieibavani et al. [12]—spanning centers in Germany, Australia, and the United States in collaboration with IBM Watson—pooled large-scale patient data to examine the impact of cohort size on model performance and generalizability. Their findings revealed that while sufficiently large datasets enhance predictive power, differences in measurement protocols and clinical practices across sites pose significant challenges, underscoring both the potential of large-scale collaborations and the need for rigorous data harmonization.

Despite the breadth of research in this field, several gaps limit the strength and generalizability of existing models. First, many published studies involve relatively small samples—sometimes fewer than 100 patients—restricting any models’ power and external validity [14,15]. Second, most models are assessed retrospectively on the same dataset used for development, or on a small, contemporaneous holdout sample [16,17]. In other words, chronologically splitting the data into training and “future” test sets to simulate real-world model performance is rarely performed. Evaluating models on a strictly later, out-of-sample cohort more closely approximates the true clinical scenario. Finally, while expert clinical judgment remains a mainstay of candidate assessment, few—if any—studies have formally compared model predictions to the prognostic accuracy of experienced clinicians. Establishing such an expert baseline could illuminate the relative contributions of data-driven approaches and human expertise, and pave the way for more effective physician–machine collaboration in clinical decision-making.

In our investigation, we address these shortcomings by training and evaluating a machine learning model using a large single-center dataset of adult patients with postlingual hearing impairment. This dataset, spanning over two decades, provides a robust foundation for building a predictive model of postoperative hearing improvement based on preoperative clinical data. We further strengthen the model evaluation by creating a chronological split of the dataset to mimic “real-world” performance and by establishing a baseline of human expert estimates for comparison. Lastly, to ensure transparent reporting and facilitate future replication, we present our work in accordance with the Transparent Reporting of a multivariable prediction model for Individual Prognosis or Diagnosis (TRIPOD) guidelines [18]. By closing these gaps and thoroughly assessing model performance, we aim to aid clinicians in more effectively identifying patients most likely to benefit from a cochlear implant, thereby improving patient counseling, resource allocation, and ultimately, patient outcomes.

## 2. Materials and Methods

### 2.1. Study Design and Data Source

This study is a retrospective, longitudinal cohort investigation conducted at the Department of Otorhinolaryngology of Hannover Medical School, Germany. The department is a tertiary care center specializing in the treatment of severe hearing disorders and performs approximately 500 cochlear implantations per year. Over 9000 patients and more than 12,500 implantations are documented in its clinical database.

Data collection for this analysis began in January 2000 and included adult patients (≥18 years) with postlingual hearing loss or deafness who underwent their first cochlear implantation. The starting date was selected to ensure that current surgical procedures and state-of-the-art implant technology were used. Only patients with at least one documented follow-up visit one to two years post-surgery were considered, capturing a time point when hearing performance is typically stable.

### 2.2. Participants

Participants were included if they:

- Were 18 years of age or older at the time of surgery;
- Had postlingual onset of hearing loss;
- Underwent their first cochlear implantation (i.e., no revision surgeries).

We excluded cases lacking a follow-up visit within the first two postoperative years or those missing critical outcome measures (see Section 2.3). After applying these criteria, **2571** cases remained in the final dataset. Because this was a retrospective study using routinely collected data, no formal written consent was required from participants. This study was exempt from ethics review according to the guidelines of the Ethics Committee of Hannover Medical School, which exempts studies of this nature, specifically because the data used were fully anonymized and collected as part of routine clinical care. Num. 1897–2013. Date: 25 July 2024. The study was conducted in accordance with the principles outlined in the Declaration of Helsinki.

### 2.3. Outcome and Predictions

#### 2.3.1. Outcome Measure

The primary outcome of interest was the **monosyllabic (MS) word recognition score**, measured on the ipsilateral (implanted) side approximately one to two years post-implantation. The MS score, expressed as a percentage from 0% (no words correctly repeated) to 100% (all words correctly repeated), is a widely used clinical measure of speech recognition and is commonly reported in predictive modeling studies [5,12]. In our center, trained audiologists perform this test using medically certified audiometry equipment at various loudness levels as part of routine postoperative follow-up. When multiple MS scores were available within the one- to two-year visit window, we used the median of those values to represent the patient’s outcome.

#### 2.3.2. Predictor Variables

We collected predictor data from the routine care database described earlier. Several of these predictors required further elaboration regarding their calculation and extraction. The preoperative best word recognition score (MS) reflected the highest test result achieved by the patient at any loudness level within the year prior to surgery, while the preoperative pure tone average (PTA) was selected similarly, averaging thresholds at 500, 1000, 2000, and 4000 Hz. The time since first implantation represented the interval between the contralateral implant surgery and the first implantation, if applicable; otherwise, it was set to zero.

These particular predictors were selected for multiple reasons. First, many of them are frequently mentioned in the literature as being correlated with postoperative hearing outcomes [5,8]. Second, consultation with domain experts confirmed their clinical relevance and feasibility. Third, the chosen predictors were consistently documented in our database, which facilitated missing-data management. Finally, because we intend for the model to be used in clinical practice with potential manual data entry by clinicians, the total number of predictors had to remain within a manageable range.

The chosen predictors, used for training all models, were:

- **Age at implantation (years)**
- **Duration of deafness (years)** on the ipsilateral side
- **Best preoperative MS score (%)** on ipsilateral and contralateral sides
  ⚬ Taken at the most favorable loudness level within up to one year prior to surgery
- **Preoperative pure tone average (PTA, dB)** on ipsilateral and contralateral sides
  ⚬ Averaged across four frequencies: 500, 1000, 2000, and 4000 Hz
- **Onset of hearing loss**, categorized as “progredient”, “acute”, or “since childhood”, encoded as a one-hot variable
- **Time since first implantation (years)**
  ⚬ Applicable only to patients who had a contralateral implant before their first implantation; otherwise, set to zero

### 2.4. Data Processing and Handling of Missing Data

No formal sample size calculation was conducted because we included all eligible cases to maximize generalizability and model robustness. Data on key outcomes (MS score) and core predictors (e.g., ipsilateral MS score, ipsilateral PTA) were mandatory for inclusion; entries missing any of these critical variables were excluded.

Most clinical audiometric measures (e.g., MS, PTA) are routinely collected at our center, so missingness in these fields was limited and assumed to be missing completely at random. Any residual missing values in other predictors were imputed using a **k-nearest neighbors** technique (k = 5) [19], which we fit exclusively on the training set to avoid data leakage into testing. Detailed information on how we split the dataset for training and testing is provided in Section 2.5.

A brief overview of patient characteristics, including distributions of age, deafness duration, and preoperative measures, is presented in Table 1. Subsequent sections detail how the training and test sets were defined to evaluate model performance under conditions that closely approximate real-world clinical usage.

### 2.5. Data Analysis and Machine Learning Methods

All predictors except “onset of hearing loss” were continuous variables and used “as is,” with no additional scaling or transformation prior to model training. The “onset of hearing loss” variable was treated as a categorical feature and **one-hot encoded**, resulting in three binary columns:

- Acute onset (1/0)
- Progredient onset (1/0)
- Onset since childhood (1/0)

We employed **a decision tree-based regression model** to predict a patient’s monosyllabic (MS) word recognition score at one to two years post-implantation [20]. Decision trees are a well-established, highly interpretable machine learning algorithm widely used for structured (tabular) clinical data. During model training, the algorithm learns a series of sequential decision rules that split the data into subsets (leaves). Each leaf is associated with a mean outcome value, which becomes the model’s prediction for any new patient whose features follow that path of decision splits.

Decision trees can capture **non-linear relationships** between predictors and the outcome yet remain **transparent**: clinicians and researchers can inspect the learned decision paths to assess their clinical plausibility. For further technical details on decision trees, see [21].

To mitigate overfitting and identify optimal hyperparameters (e.g., tree depth, minimum samples per leaf), we utilized **k-fold cross-validation (CV)** [22]. Specifically, we employed a **grouped 10-fold CV** approach to ensure that the same patient (e.g., someone implanted on both sides) never appeared in both the training and validation folds. In each fold, the model was trained on k-1 subsets and validated on the remaining subset, thereby providing an unbiased assessment of the model’s predictive performance on unseen data.

To assess prediction accuracy, we primarily used **mean absolute error (MAE)** to evaluate how closely the predicted MS scores matched the actual MS scores in the validation and test sets. The *MAE* is computed as:

$$MAE=\frac{\sum_{i=1}^{n}\left|y_{i}-x_{i}\right|}{n}$$

where *y_i_* = true value, *x_i_* = prediction, and *n* = total number of data points.

A mean absolute error of zero indicates perfect agreement between predictions and reality. In addition, we examined observed vs. predicted plots to visualize the distribution of model errors.

We established three separate test datasets to gain a comprehensive understanding of the model’s real-world performance:

1. **Random Test Split (10%)**: A simple random sample comprising 10% of the overall dataset, set aside before model training.
2. **Chronologically New Data**: To approximate real-life usage where future patients may differ from those on whom the model was trained, we created a “future” dataset containing cases from 2020 onward, ensuring these were not included in the training set. This dataset was to evaluate if the model, trained on older data, would still be able to predict performance of more recently implanted patients.
3. **Expert Comparison Dataset**: We prospectively collected 19 cases for which experienced audiologists at our center provided predicted MS scores. This enabled a direct comparison of model-based predictions against human expert estimations on the same individuals. The dataset includes all relevant predictors, the actual postoperative MS score (ground truth), and the audiologist’s predicted MS score.

### 2.6. Implementation

All data analysis steps—missing value imputation, feature engineering, model development, and performance evaluation—were implemented in **Python 3.9.7** (Python Software Foundation, Wilmington, DE, USA). We used the open-source libraries **Pandas** (v1.4.2) for data manipulation [23], **Scikit-learn** (v1.2.dev) for machine learning algorithms [24] and **Matplotlib** (v3.5.1) for data visualization [25].

These tools facilitated efficient preprocessing, cross-validation, hyperparameter tuning, and model deployment workflows.

## 3. Results

Following established guidelines for reporting clinical prediction models that utilize regression or machine learning methods [18], we present the results in three sections: the flow of participants through the study, extended patient characteristics, and the outcomes of model development, specification, and performance.

### 3.1. Participant Flow and Dataset Preparation

**Figure 1:**
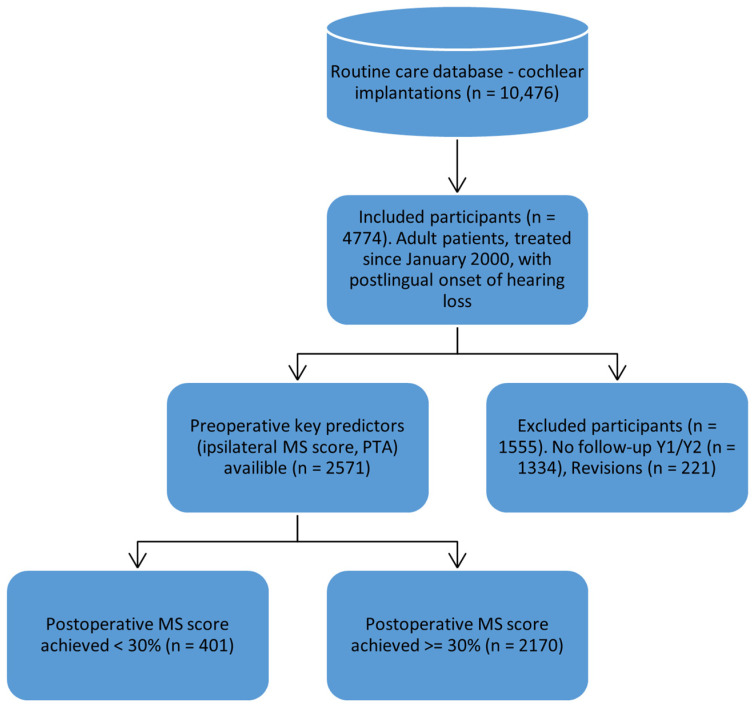
Flow chart of the study participants.

Figure 1 illustrates the flow of participants throughout the study, beginning with the entire dataset from the clinical database. The flowchart details the application of inclusion criteria and the exclusion of cases that did not meet study requirements. The final nodes of the diagram display the distribution of patients achieving “poor” versus “good” monosyllabic (MS) word recognition scores after cochlear implantation.

Model development and evaluation followed standard machine learning practices. After final data preparation, we used the complete dataset for training and evaluation without further adjustments. A random 90% of the data was allocated for model training and hyperparameter tuning. The training dataset is described in Table 2. Hyperparameters for the final model were selected based on cross-validation performance (as described in the Materials and Methods Section). Specifically, we found that a Decision Tree Regressor performed best when trained using the absolute error criterion, a maximum tree depth of 5, and a minimum of 5 samples required for splitting at a leaf node.

After determining optimal hyperparameters, we trained and evaluated the model using all three test datasets:

1. A **random split test set** comprising the remaining 10% of the data.
2. A **chronologically new test set** containing patients treated after 2020.
3. An **expert estimation test set**, created to compare model predictions with those of experienced healthcare professionals, i.e., audiologists.

To improve outcome variability, the final test results represent the averaged predictions from 10 different models trained during the cross-validation process (see Section 2).

### 3.2. Model Performance

With all data preparation steps clarified and participant characteristics outlined, we report the performance of the model across the test datasets and provide visualizations to support detailed performance analysis.

**Figure 2:**
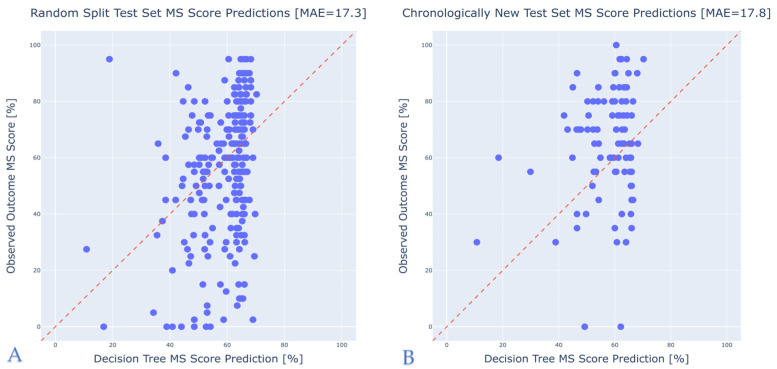
Observed vs. predicted model performance on test sets. (**A**) Random split test set. (**B**) Chronologically new test set.

On the random split test set, the model achieved a mean absolute error (MAE) of 17.3%, with a standard deviation (STD) of 14.3%. On the chronologically new test set, the model achieved an MAE of 17.8% with a standard deviation of 12.4%. These results indicate relatively low prediction error across both datasets. The consistent performance on the chronologically new dataset suggests the model has potential for real-world application, as it shows no significant performance degradation when tested on future data. Scatter plots of observed versus predicted MS scores for both datasets are presented in Figure 2, highlighting the model’s ability to estimate whether patients will achieve “poor” or “good” postoperative performance.

### 3.3. Comparison with Expert Predictions

**Figure 3:**
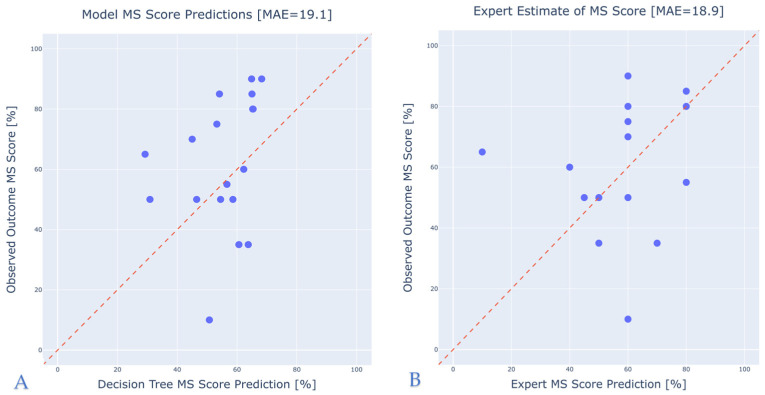
Model vs. expert comparison scatter plots. (**A**) Decision tree prediction. (**B**) Expert estimation.

The model’s predictive performance was further evaluated using the **expert estimation test set**, which included predictions provided by experienced audiologists with access to the full medical records of the patients. On this dataset, the model achieved an MAE of 19.1%, while the expert predictions achieved an MAE of 18.9%. These results demonstrate that the model’s performance is comparable to that of domain experts and operates within a clinically reasonable prediction range. Figure 3 displays scatter plots comparing observed versus predicted scores for both the model and the expert estimations.

## 4. Discussion

Our model establishes relationships between preoperative demographic, audiological, and other clinical data and postoperative cochlear implant (CI) performance using decision trees. This well-established and robust machine learning algorithm is particularly suited to capturing non-linear relationships, which is invaluable when addressing the complex and often non-linear nature of patient outcomes in cochlear implant surgery. Unlike linear models that assume a direct proportionality between inputs and outcomes, decision trees can partition data into segments where different rules apply. This flexibility reflects the variability observed in clinical settings and allows the model to account for diverse patient responses to cochlear implantation. Such non-linearity is critical for analyzing our dataset, where individual variability defies simplistic, linear assumptions.

While the predictive performance of our model is comparable to previous studies, it is noteworthy that this result was achieved using the largest single-center dataset described in the scientific literature to date, which should theoretically confer a significant advantage in terms of homogeneity and specificity. This homogeneity within the single-site dataset enables our model to achieve a high level of internal consistency, making it particularly well-suited for use in clinical environments with similar patient populations and practices. In contrast, Shafieibavani et al. [12] employed a larger cohort drawn from three international settings, which, while increasing the sample size, might have compromised data consistency due to varying clinical practices and patient demographics. Nonetheless, advancing the development of more accurate and robust models will inevitably require the pooling of data across multiple clinics and institutions to achieve sufficient sample sizes and broader generalizability.

To address the challenges posed by variability in testing methodologies across audiological centers, efforts are underway to harmonize the interpretability of different audiological tests, enabling the pooling of data from diverse setups. For example, recent work by Buhl et al. [26] demonstrates a promising approach by developing a model-based procedure to estimate speech recognition thresholds (SRTs) from existing clinical data, such as monosyllabic word recognition scores and audiograms. By leveraging the psychometric relationship between speech test outcomes and discrimination loss, their method provides a framework for integrating data from disparate sources, even when patient records are incomplete. Saak et al. [27] have explored the use of auditory profiles—a framework that categorizes patients into distinct groups based on the extent and nature of their hearing impairment. These profiles act as an intermediary between raw patient data and the input vectors used in machine learning models, offering a way to standardize heterogeneous data. However, while auditory profiles may help mitigate inconsistencies, they introduce an additional layer of abstraction that risks diluting the granularity and predictive power of the original data.

Thus, the ultimate goal must remain the standardization of clinical procedures and data structures across sites. While some medical fields are advancing or have already established agreed-upon standards for procedures and data, audiology still lags in this aspect, though efforts are being made to bridge this gap. An international group of experts, including our team, is actively developing open data standards to advance data-driven audiology. These efforts focus on standardizing data formats for cross-institutional data exchange, guided by the FAIR principles (Findability, Accessibility, Interoperability, Reusability), emphasizing interoperability. One promising approach is the use of openEHR, an interoperability standard that unambiguously describes and stores clinical concepts, both syntactically and semantically. Recognizing the importance of community involvement, a community-driven approach is being pursued to ensure that these open data standards are broadly usable and widely adopted. In May 2023, the EFAS (European Federation of Audiology Societies) International Working Group on Standardized Data Formats for Big Data in Audiology was established to coordinate these activities. The archetypes developed to date are publicly available in the Clinical Knowledge Manager (CKM) of the Highmed consortium [28].

Being able to pool data across different institutions or clinics, maybe even across borders and languages using the appropriate testing material, large scale data analyses could be performed, enabling the application of up-to-date big data machine learning approaches.

Our current single-site data model was validated using two subsets of our internal data: one a random sample, and another reflecting recent patient data to simulate real-life application scenarios. This dual validation not only assesses model performance but also offers insight into its practical utility in clinical settings. While internal validation might limit broader generalizability, it is important to note that our model’s primary use case might be within a specific care center or network rather than a global context, making local validation particularly relevant. A distinctive aspect of our study is the introduction of a human expert baseline, marking the first use of a domain expert prediction dataset in this field. Though constrained in size, this baseline sets a new standard for evaluating model performance against human expertise, highlighting the nuanced advantages of decision trees when applied to complex medical data.

The data from our study also challenge the common assumption within the cochlear implantation field that seasoned clinicians, with their ‘gut-feeling’, can predict outcomes more accurately than statistical models. Our findings indicate that human predictions do not outperform our model’s predictions; in fact, there was a slight trend suggesting that our model might predict outcomes more accurately. This observation is supported by recent research from Philpott et al. [29], which highlighted that even when experts are provided with detailed candidate data, they struggle to predict individual outcomes, often defaulting to predicting an average score typically seen in cochlear implant patients. This suggests that while clinical intuition is valuable, it may not suffice for nuanced, patient-specific predictions where machine learning models, like ours, can offer more precise forecasts by systematically analyzing complex datasets.

It is crucial to acknowledge the limitations of our study. Firstly, our model was both trained and evaluated using retrospectively collected data. Given that our routine care database has evolved over decades, changes in methodology for assessing and inputting predictor data could have occurred, potentially introducing data drift and complicating pattern recognition for our models. Secondly, by training and evaluating our model with data from a single center, we might have limited the model’s generalizability. External validation is a critical step in model development that assesses how well a model can generalize to other populations, and we recognize this as an area for future improvement. Specifically, our model might be most applicable for further testing in German hearing clinics, where clinical practices and regulations are similar. Caution should be exercised when applying the model to different geographical populations, necessitating careful adjustments and further validation. Lastly, while we chose the monosyllabic test as our prediction outcome due to its routine use and availability, this test has its own limitations, including high test-to-test variability, which could impact the reliability of our model’s predictions.

Still, we think that our predictive model has the potential to aid in clinical decision-making by providing rough estimates of benefits for cochlear implant candidates. It utilizes clinically relevant predictors commonly measured in many settings, and our web application is user-friendly, requiring only a minimal increase in assessment time. However, we are not yet advocating for its use in clinical practice. Advancing to that stage would require extensive further investigation through a carefully designed randomized clinical trial.

## Figures and Tables

**Table 1:** Key study characteristics.

Characteristic	Train/Test Set (*n* = 2479)	“Recent” Test Set (*n* = 92)	Expert Estimation (*n* = 18)
Cochlear Implantation Period	2000–2019	2020–2022	2022–2023
Study Design	Retrospective Longitudinal Cohort	Retrospective Longitudinal Cohort	Retrospective Longitudinal Cohort with Prospectively Collected Expert Estimates
Setting	Tertiary Care Center in Large University Hospital (Hannover, Germany)		
Inclusion Criteria	Adult patients with severe hearing loss/deafness treated with cochlear implantation		Same + postoperative monosyllabic score being estimated by expert
Outcome	Monosyllabic score on implanted side in 1 year after surgery		
Average Postoperative MS score (std), %	55 (25)	65 (20)	60 (20)
Average Age at Implantation (range), y	59 (18–94)	61 (23–93)	63 (18–86)
Average Preoperative MS score, ipsilateral (std), %	16 (23)	18 (22)	23 (24)
Average Preoperative PTA, ipsilateral, (std), dB	102 (20)	97 (19)	95 (23)

**Table 2:** Extended participants characteristics (final dataset).

Characteristic	All Patients (*n* = 2479)	MS < 30% (*n* = 399)	MS ≥ 30% (*n* = 2080)
Median Age (IQR), y	60 (49–72)	63 (50–74)	60 (49–71)
Median MS Score Ipsilateral (IQR), %	0 (0–30)	0 (0–15)	5 (0–30)
Median MS Score Contralateral (IQR), %	45 (0–85)	40 (0–85)	45 (0–80)
Median PTA Ipsilateral (IQR), dB	102 (85–120)	110 (90–130)	101 (85–118)
Median PTA Contralateral (IQR), dB	82 (61–110)	81 (56–107)	83 (62–111)
Median Duration of Deafness (IQR), y	1.7 (0–8.2)	4.4 (0.7–19.5)	1.5 (0–6.8)
Progredient Onset (% of cases), *n* cases	2006 (80)	296 (74)	1710 (82)
Acute Onset (% of cases), *n* cases	408 (16)	92 (23)	315 (15)
Onset Since Childhood (% of cases), *n* cases	83 (4)	15 (3)	68 (3)

MS = monosyllabic score, IQR = interquartile range.

## Data Availability

Data will be available upon reasonable request from the corresponding author.

## References

[1] Olusanya B.O., Neumann K.J., Saunders J.E. (2014). The Global Burden of Disabling Hearing Impairment: A Call to Action. Bull. World Health Organ..

[2] World Report on Hearing. https://www.who.int/publications/i/item/9789240020481.

[3] Lin F.R., Yaffe K., Xia J., Xue Q.-L., Harris T.B., Purchase-Helzner E., Satterfield S., Ayonayon H.N., Ferrucci L., Simonsick E.M. (2013). Hearing Loss and Cognitive Decline in Older Adults. JAMA Intern. Med..

[4] Gifford R.H., Shallop J.K., Peterson A.M. (2008). Speech Recognition Materials and Ceiling Effects: Considerations for Cochlear Implant Programs. Audiol. Neurootol..

[5] Boisvert I., Reis M., Au A., Cowan R., Dowell R.C. (2020). Cochlear Implantation Outcomes in Adults: A Scoping Review. PLoS ONE.

[6] Pisoni D.B., Kronenberger W.G., Harris M.S., Moberly A.C. (2017). Three Challenges for Future Research on Cochlear Implants. World J. Otorhinolaryngol.—Head Neck Surg..

[7] Lazard D.S., Giraud A.L., Truy E., Lee H.J. (2011). Evolution of Non-Speech Sound Memory in Postlingual Deafness: Implications for Cochlear Implant Rehabilitation. Neuropsychologia.

[8] Velde H., Rademaker M., Damen J., Smit A., Stegeman I. (2021). Prediction Models for Clinical Outcome after Cochlear Implantation: A Systematic Review. J. Clin. Epidemiol..

[9] Holden L.K., Finley C.C., Firszt J.B., Holden T.A., Brenner C., Potts L.G., Gotter B.D., Vanderhoof S.S., Mispagel K., Heydebrand G. (2013). Factors Affecting Open-Set Word Recognition in Adults With Cochlear Implants. Ear Hear..

[10] Hoppe U., Hast A., Hornung J., Hocke T. (2023). Evolving a Model for Cochlear Implant Outcome. J. Clin. Med..

[11] Crowson M.G., Dixon P., Mahmood R., Lee J.W., Shipp D., Le T., Lin V., Chen J., Chan T.C.Y. (2020). Predicting Postoperative Cochlear Implant Performance Using Supervised Machine Learning. Otol. Neurotol..

[12] Shafieibavani E., Goudey B., Kiral I., Zhong P., Jimeno-Yepes A., Swan A., Gambhir M., Buechner A., Kludt E., Eikelboom R.H. (2021). Predictive Models for Cochlear Implant Outcomes: Performance, Generalizability, and the Impact of Cohort Size. Trends Hear..

[13] Kim H., Kang W.S., Park H.J., Lee J.Y., Park J.W., Kim Y., Seo J.W., Kwak M.Y., Kang B.C., Yang C.J. (2018). Cochlear Implantation in Postlingually Deaf Adults Is Time-Sensitive Towards Positive Outcome: Prediction Using Advanced Machine Learning Techniques. Sci. Rep..

[14] Roditi R.E., Poissant S.F., Bero E.M., Lee D.J. (2009). A Predictive Model of Cochlear Implant Performance in Postlingually Deafened Adults. Otol. Neurotol..

[15] Plant K., McDermott H., van Hoesel R., Dawson P., Cowan R. (2016). Factors Predicting Postoperative Unilateral and Bilateral Speech Recognition in Adult Cochlear Implant Recipients with Acoustic Hearing. Ear Hear..

[16] Favaretto N., Marioni G., Brotto D., Sorrentino F., Gheller F., Castiglione A., Montino S., Giacomelli L., Trevisi P., Martini A. (2019). Cochlear Implant Outcomes in the Elderly: A Uni- and Multivariate Analyses of Prognostic Factors. Eur. Arch. Otorhinolaryngol..

[17] James C.J., Karoui C., Laborde M.-L., Lepage B., Molinier C.-É., Tartayre M., Escudé B., Deguine O., Marx M., Fraysse B. (2019). Early Sentence Recognition in Adult Cochlear Implant Users. Ear Hear..

[18] Collins G.S., Moons K.G.M., Dhiman P., Riley R.D., Beam A.L., Van Calster B., Ghassemi M., Liu X., Reitsma J.B., Van Smeden M. (2024). TRIPOD+AI Statement: Updated Guidance for Reporting Clinical Prediction Models That Use Regression or Machine Learning Methods. BMJ.

[19] Troyanskaya O., Cantor M., Sherlock G., Brown P., Hastie T., Tibshirani R., Botstein D., Altman R.B. (2001). Missing Value Estimation Methods for DNA Microarrays. Bioinformatics.

[20] Breiman L., Friedman J., Olshen R.A., Stone C.J. (2017). Classification and Regression Trees.

[21] Hastie T., Tibshirani R., Friedman J., Hastie T., Tibshirani R., Friedman J. (2009). Additive Models, Trees, and Related Methods. The Elements of Statistical Learning: Data Mining, Inference, and Prediction.

[22] Kohavi R. (2001). A Study of Cross-Validation and Bootstrap for Accuracy Estimation and Model Selection. IJCAL.

[23] Mckinney W. Data Structures for Statistical Computing in Python. Proceedings of the 9th Python in Science Conference.

[24] Pedregosa F., Varoquaux G., Gramfort A., Michel V., Thirion B., Grisel O., Blondel M., Prettenhofer P., Weiss R., Dubourg V. (2011). Scikit-Learn: Machine Learning in Python. J. Mach. Learn. Res..

[25] Hunter J.D. (2007). Matplotlib: A 2D Graphics Environment. Comput. Sci. Eng..

[26] Buhl M., Kludt E., Schell-Majoor L., Avan P., Campi M. (2025). Discrimination Loss vs. SRT: A Model-Based Approach towards Harmonizing Speech Test Interpretations. arXiv.

[27] Saak S., Huelsmeier D., Kollmeier B., Buhl M. (2022). A Flexible Data-Driven Audiological Patient Stratification Method for Deriving Auditory Profiles. Front. Neurol..

[28] Systems S.G. Ocean Health Clinical Knowledge Manager. https://ckm.highmed.org/ckm/projects/1246.152.56.

[29] Philpott N., Philips B., Donders R., Mylanus E., Huinck W. (2024). Variability in Clinicians’ Prediction Accuracy for Outcomes of Adult Cochlear Implant Users. Int. J. Audiol..

